# Inhibition of the ISR abrogates mGluR5-dependent long-term depression and spatial memory deficits in a rat model of Alzheimer’s disease

**DOI:** 10.1038/s41398-022-01862-9

**Published:** 2022-03-08

**Authors:** Zhengtao Hu, Pengpeng Yu, Yangyang Zhang, Yin Yang, Manyi Zhu, Shuangying Qin, Ji-Tian Xu, Dongxiao Duan, Yong Wu, Deguo Wang, Michael J. Rowan, Neng-Wei Hu

**Affiliations:** 1 Department of Physiology and Neurobiology, School of Basic Medical Sciences, Zhengzhou University, 100 Science Avenue, Zhengzhou, 450001 China; 2 Department of Gerontology, The First Affiliated Hospital of Wannan Medical College, Wuhu, 241001 China; 3 Key Laboratory of Non-coding RNA Transformation Research of Anhui Higher Education Institution, Wannan Medical College, Wuhu, Anhui 241001 China; 4 Department of Pharmacology &Therapeutics and Institute of Neuroscience, Trinity College, Dublin, 2 Ireland

**Keywords:** Learning and memory, Diseases

## Abstract

Soluble amyloid-β-protein (Aβ) oligomers, a major hallmark of AD, trigger the integrated stress response (ISR) via multiple pathologies including neuronal hyperactivation, microvascular hypoxia, and neuroinflammation. Increasing eIF2α phosphorylation, the core event of ISR, facilitates metabotropic glutamate receptor (mGluR)-dependent long-term depression (LTD), and suppressing its phosphorylation has the opposite effect. Having found the facilitation of mGluR5-LTD by Aβ in live rats, we wondered if suppressing eIF2α phosphorylation cascade would protect against the synaptic plasticity and cognitive disrupting effects of Aβ. We demonstrate here that the facilitation of mGluR5-LTD in a delayed rat model by single i.c.v. injection of synthetic Aβ_1–42_. Systemic administration of the small-molecule inhibitor of the ISR called ISRIB (trans-isomer) prevents Aβ-facilitated LTD and abrogates spatial learning and memory deficits in the hippocampus in exogenous synthetic Aβ-injected rats. Moreover, ex vivo evidence indicates that ISRIB normalizes protein synthesis in the hippocampus. Targeting the ISR by suppressing the eIF2α phosphorylation cascade with the eIF2B activator ISRIB may provide protective effects against the synaptic and cognitive disruptive effects of Aβ which likely mediate the early stage of sporadic AD.

## Introduction

In the brains of patients with Alzheimer’s disease (AD), elements of cellular stress termed the “integrated stress response” (ISR) and its initiators are persistently abnormal [1, 2], with translational dysregulation due to aberrant phosphorylation of the eukaryotic initiation factor 2 α-subunit (eIF2α) being well documented [3–5]. Microvascular hypoxia, neuronal hyperactivation, neuroinflammation, and the accumulation of unfolded proteins in the endoplasmic reticulum can be triggered by soluble amyloid-β-protein (Aβ) oligomers, a major hallmark of AD. These Aβ-triggered pathologies are common inducers of the ISR [2].

Synaptic plasticity disruption is a core feature of models of early AD [6]. Soluble Aβ induces impairment of hippocampal long-term potentiation (LTP) and facilitation of long-term depression (LTD) [7]. In contrast to LTP, much less is known about the mechanisms and functions underlying LTD [8].

It is well established that protein synthesis is important for memory formation and synaptic plasticity including LTD [1]. Although accumulating evidence suggests that LTD serves as a learning and memory mechanism in the mammalian brain [9], certain forms of LTD, such as metabotropic glutamate receptor (mGluR)-dependent LTD, are enhanced in models of different neurological diseases including AD [10, 11]. Although the cellular mechanisms of hippocampal mGluR-LTD have been documented, little is known about the contribution of this form of plasticity to hippocampal-dependent learning. mGluR-LTD has been associated with impaired cue and spatial discrimination in the Morris water maze (MWM) in aged Fischer 344 rats [12]. Chronic pharmacological mGluR5 antagonist treatment has been reported to improve performance in the MWM in an AD mouse model over expressing Aβ [13].

Since increasing eIF2α phosphorylation facilitates mGluR-LTD [14] and suppressing its phosphorylation has the opposite effect [14–17], we wondered if suppressing eIF2α phosphorylation cascade would protect against the synaptic plasticity and cognitive disrupting effects of Aβ.

Of particular potential therapeutic value, a brain-penetrant small-molecule ISR inhibitor, called ISRIB (trans-isomer), which restores translation downstream of kinase phosphorylation of eIF2α, by activating the nucleotide exchange factor eIF2B [18–22], has been found to have beneficial effects in neurodegeneration models [23] and aging animals [24], but without the pancreatic toxicity of PERK inhibitors, presumably, because its action is state-dependent [25].

To determine whether ISRIB may be beneficial in early sporadic AD, we chose to study the disruptive effects of injecting exogenous Aβ, a hall mark of the disease [26]. We examined the effects of ISRIB both on LTD facilitation and the persistent behavioral deficits in the MWM triggered by a single injection of synthetic Aβ_1–42_, a well-established model of cognitive impairment in early sporadic AD [27–30]. ISRIB abrogated both Aβ-facilitated LTD and learning and memory deficits. Moreover, we found evidence indicating that ISRIB restored aberrant low levels of general protein synthesis and high levels of transcription factor 4 (ATF4) in the hippocampus of exogenous Aβ_1–42_-injected rats. Suppressing the eIF2α phosphorylation cascade may underlie the beneficial effects of ISRIB on Aβ-mediated synaptic plasticity disruption and spatial learning and memory deficits in adult male rats.

## Materials and methods

### Animals

Animal care and experimental protocols followed the ARRIVE (Animal Research: Reporting of In Vivo Experiments) guidelines 2.0 [31] and were approved by the Animal Care and Use Committee of Zhengzhou University and Wannan Medical College, China. All efforts were made to minimize the number of animals used and their suffering.

Adult (250–350 g, 8–11 weeks old) male Wistar rats were provided by the Laboratory Animal Center of Zhengzhou University and Nanjing University. The animals were housed under a 12 h light-dark cycle at room temperature (19–22 °C).

### Intracerebroventricular administration of Aβ

For intracerebroventricular (i.c.v.) injection of synthetic Aβ, the animals were anaesthetized with ketamine (80 mg/kg, i.p.) and xylazine (8 mg/kg, i.p.) and placed in a stereotaxic apparatus. Analgesic meloxicam (1 mg/kg, s.c.) was administrated before surgery and one injection each day for three consecutive days post-surgery. The body temperature of the rats was maintained at 37–38 °C with a feedback-controlled heating blanket. Lignocaine (0.3 ml, 1% adrenaline, s.c.) was injected over the area of the skull where the holes were drilled for i.c.v. injection. After exposing the skull, two holes were drilled above the lateral ventricle with the coordinates from bregma: AP: −0.5; ML: ±1.5; DV: −4.5. The solutions (soluble Aβ_1–42_ or reverse control Aβ_42–1_) were injected in a 10 μL volume over a 6-min period with Hamilton syringe bilaterally.

The animals were monitored until full consciousness was regained and housed singly for one week or until wound healing had completed, after which they were housed in pairs with continuous access to food and water ad libitum.

### Electrophysiology

Prior to the electrophysiology experiments, animals were re-anaesthetized with urethane (1.5–1.6 g/kg, i.p.). Lignocaine (0.6 ml, 1% adrenaline, s.c.) was injected over the area of the skull where electrodes and screws were to be implanted. The body temperature of the rats was maintained at 37–38 °C with a feedback-controlled heating blanket during the whole period of recording.

Electrodes were made and implanted as described previously [11]. Briefly, monopolar recording electrodes were constructed from Teflon-coated tungsten wires (75 μm inner core diameter, 112 μm external diameter) and twisted bipolar stimulating electrodes were constructed from Teflon-coated tungsten wires (50 μm inner core diameter, 75 μm external diameter) separately. Field excitatory postsynaptic potentials (EPSPs) were recorded from the stratum radiatum in the CA1 area of the right hippocampus in response to stimulation of the ipsilateral Schaffer collateral-commissural pathway. Electrode implantation sites were identified using stereotaxic coordinates relative to bregma, with the recording site located 3.4 mm posterior to bregma and 2.5 mm lateral to midline, and the stimulating site 4.2 mm posterior to bregma and 3.8 mm lateral to midline. The final placement of electrodes was optimized by using electrophysiological criteria and confirmed via postmortem analysis.

Test EPSPs were evoked by a single square wave pulse (0.2 ms duration) at a frequency of 0.033 Hz and an intensity that triggered a 50% maximum EPSP response. A relatively weak LFS protocol, used to study the Aβ-mediated facilitation of LTD, consisted of 300 pulses (0.2 ms duration) at 1 Hz, with an intensity that evoked 95% maximum amplitude. None of the conditioning stimulation protocols elicited any detectible abnormal changes in background EEG, which was recorded from the hippocampus throughout the experiments.

### Morris water maze

Based on a previously published protocol [29], two weeks after a single i.c.v. injection of soluble Aβ_1–42_ or reverse control Aβ_42–1_ under-recovery anesthesia, the rats were trained in a water pool (150 cm diameter) with a hidden platform of 10 cm diameter. Animals were handled daily for 3 days before the experiment and then trained according to one of two protocols. The standard training protocol consisted of four swimming trials per day whereas a relatively weak protocol consisted of one swimming trial per day. Each animal swam until it found the hidden platform or 120 s, when it was gently guided to the platform and stayed there for 10 s before being returned to the cage. Immediately after the last daily training trial, the animals were injected intraperitoneally with ISRIB (0.25 mg/kg) as reported [18]. To investigate the persistent beneficial effects after ceasing injection of ISRIB in rats undergoing the weak training protocol, a one-week break was given after training day 5, when a clear improvement in the escape latency of Aβ_1–42_-injected rats was present. For the probe test, the platform was removed and each animal was allowed to swim for 120 s, while its swimming trajectory was monitored with a video tracking system (Smart, PANLAB, Spain). At the end of the probe test, the animals were killed by decapitation. The brains of rats that underwent the standard training protocol were used in the western blot study.

### Western blot

The whole brain was taken out and the hippocampus from both sides was separated and frozen immediately in liquid nitrogen and stored at −80 °C. The tissues were homogenized in lysis buffer (10 mM Tris-HCl, pH 7.5, 150 mM NaCl, and 0.5% Triton X-100, 0.1 mM PMSF) containing 1% protease inhibitor Cocktail (Sigma-Aldrich, CW2200S) and 1% phosphatase inhibitor Cocktail (Sigma-Aldrich, CW2383S). The protein concentrations were determined by the BCA Protein Assay Kit (Solarbio, PC0020). Thirty μg of total protein was loaded in each well and samples were separated by 10% Tris-glycine SDS-PAGE. The proteins were transferred onto polyvinylidene fluoride (PVDF) membranes (Millipore, IPVH00010). Then the membranes were blocked with 5% non-fat milk for 60 min at room temperature. After blocking, the membranes were incubated respectively with the following primary antibodies, rabbit anti-ATF4 (Abcam, ab23760, 1:1000; ABclonal, A18687, 1:1000) and rabbit anti-GAPDH (ABclonal, AC001, 1:1000) overnight at 4 °C. After primary antibody incubation, the membranes were washed three times in TBST and then incubated with HRP-conjugated goat anti-rabbit IgG (Jackson ImmunoResearch, 111-035-144, 1:10000) for 2 h at room temperature. Finally, the target protein bands were visualized with chemiluminescence reagents (Affinity, KF005) and then detected with ProteinSimple System (Hybrid HY8300, FluorChem E system, USA). Quantification of the protein expression was calculated with ImageJ (version 1.52a). For the detection of the phosphorylation of eIF2α, the PVDF membranes were first incubated with rabbit anti-phospho-eIF2α (Ser51) (Abcam, ab32157, 1:500) and the corresponding bands were detected. Then, the primary antibodies were stripped with strip buffer (200 mM Glycine, 3.5 mM SDS, 1% Tween-20, pH 2.1). After stripping, the membrane was re-blocked and incubated with rabbit anti-total eIF2α antibody (ABclonal, A0764, 1:1000).

### Synthetic Aβ

Wild-type full-length Aβ_1–42_ and reverse control Aβ_42–1_ (ChinaPeptides, Shanghai) were prepared at a nominal concentration of 100 µM by dissolving known weights of peptides in mild alkali (0.1% ammonium hydroxide) in milliQ water to avoid isoelectric precipitation. Both solutions were then centrifuged at 100,000 × *g* for 3 h, which readily pellets fibrils and protofibrils, and the upper 75% of the supernatant taken. An aliquot of the supernatant was reserved to estimate the relative peptide concentration using the micro BCA protein assay (Thermo-Fisher Scientific Life Science Research Products, Rockford, IL). Then the concentrations of the remaining supernatants of Aβ_1–42_ and reverse control Aβ_42–1_ were adjusted to 75 µM stock solutions and stored at −80 °C until required.

### Pharmacological agents

Trans-N,N′-(Cyclohexane-1,4-diyl)bis(2-(4-chlorophenoxy) acetamide (ISRIB, Sigma, SML0843) was dissolved in dimethyl sulfoxide (DMSO) with gentle warming and diluted in polyethylene glycol 400 (PEG400) or saline before injection; 1:1 DMSO and PEG400 or 1% v/v solution of DMSO in saline was used as vehicle control. The choice of dose and timing of ISRIB administration was based on previous reports [14, 17, 18, 24, 32–34] and our study of the pharmacokinetics of ISRIB in live rats (see below). 4-(3-phosphonopropyl)piperazine-2-carboxylic acid ((±)-CPP, Alomone, C-175) and 3-((2-methyl-1,3-thiazol-4-yl)ethynyl)pyridine hydrochloride (MTEP hydrochloride, Abcam, ab120035) were prepared in distilled water and diluted with saline to the required concentration.

### Pharmacokinetics of ISRIB

ISRIB was dissolved in DMSO then diluted 1:1 in Super-Refined PEG 400. Intraperitoneal (i.p.) injection was performed on 8–11-week-old male Wistar rats (Nanjing University, Nanjing, China). Animals received a single injection at two different doses (0.25 mg/kg and 2.5 mg/kg) in groups of three rats. Blood (100 μl) was collected from the saphenous vein at intervals post-dosing (2, 6, 24 h) in EDTA containing collection tubes and plasma was prepared for analysis after centrifugating the blood samples at 12,000 RPM for 10 min. The concentration of ISRIB was detected by high-performance liquid chromatography (HPLC).

### SUnSET

Surface sensing of translation (SUnSET) assay was performed as previously described [35, 36]. Soluble Aβ_1–42_ or reverse sequence control peptide Aβ_42–1_ was injected (i.c.v., 10 μL each side, 75 μM) under isoflurane anesthesia. All the rats were singly housed after full recovery from anesthesia. Twenty-four hours after Aβ injection, the rats were re-anesthetized with isoflurane and puromycin (100 μg/10 μL, i.c.v., 5 μL each side) was injected to label the nascent polypeptides. Rats were sacrificed 2 h after puromycin administration and the hippocampi were collected immediately. The tissue was homogenized and the total protein was extracted with RIPA buffer. Then the proteins were separated with SDS-PAGE and transferred to PVDF membrane. Puromycin was detected with anti-puromycin antibody (Millipore, MABE343, 1:25,000). After HRP-conjugated secondary antibody incubation, the bands were detected with ECL method. ISRIB (2.5 mg/kg, i.p.) or vehicle was administrated about 1 h before Aβ_1–42_ or Aβ_42–1_ injection.

### Data analysis

Brown-Forsythe test was used to evaluate the similarities of variances among the groups. All data were normally distributed. Values are expressed as the mean ± s.e.m. For the electrophysiology experiments, the last 10 min prior to LFS was used to calculate the “Pre” –induction EPSP amplitude. Unless otherwise stated the magnitude of LTD was measured over the last 10 min at 90 min after (“Post”) LFS. No data were excluded, and control experiments were interleaved randomly throughout. To compare between groups of three or more, one-way ANOVA with Bonferroni multiple comparisons was used. A two-tailed paired Student’s *t*-test (paired *t*) was used to compare between “Pre” and “Post” within groups. For the Morris water maze test, two-way ANOVA followed by a post hoc Bonferroni multiple comparisons test was used for escape latency analysis and one-way ANOVA with Bonferroni multiple comparisons was used to analyze the results from the probe trials. For the Western blots and SUnSET, one-way ANOVA with Bonferroni multiple comparisons was used. The experiments of Western blots were performed blind to treatment conditions. A value of *P* < 0.05 was considered statistically significant (*P < 0.05, **P < 0.01, ***P < 0.001, ****P < 0.0001).

## Results

### In vivo LTD facilitation in exogenous Aβ-injected rats is mGluR5-dependent

Previously we reported that Aβ usurps normal mechanisms of LTD at glutamatergic synapses between CA3 and CA1 hippocampal pyramidal neurons in the acutely anaesthetized adult rat [11], here we investigated the disruption of LTD in a delayed animal model by single i.c.v. injection of synthetic Aβ_1–42_, a well-established model of cognitive impairment in early sporadic AD [27–30].

**Fig. 1:**
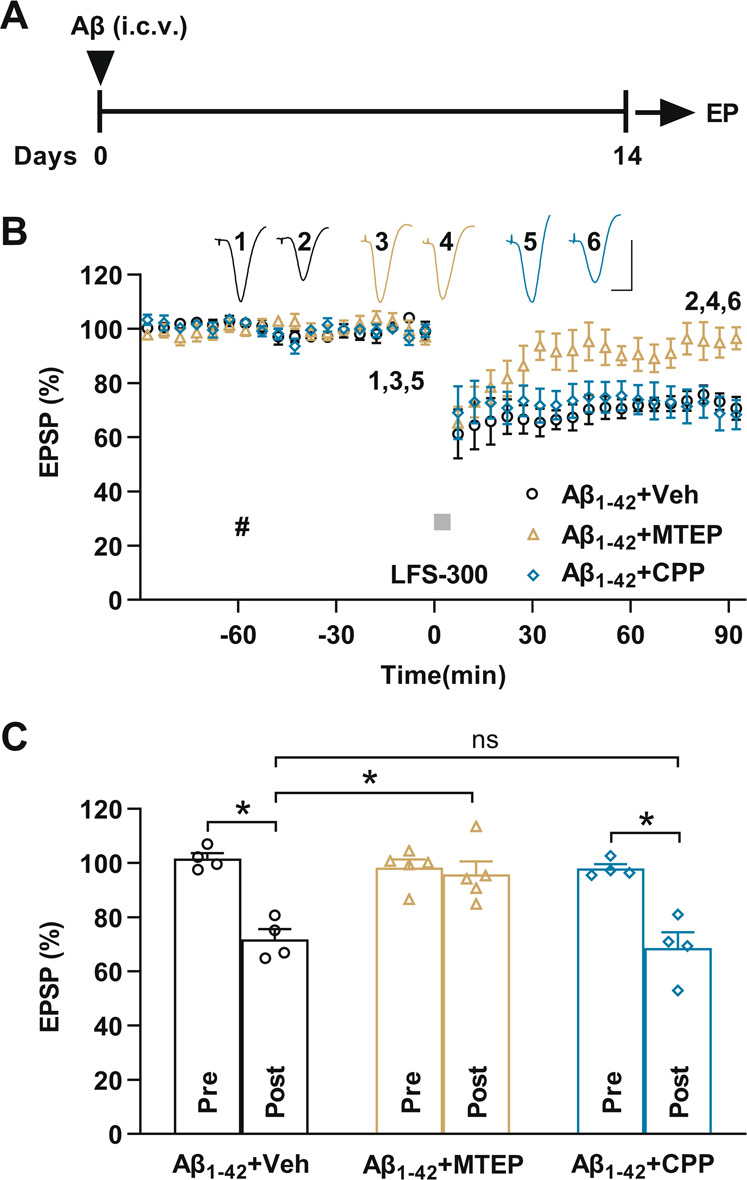
mGluR5-dependent LTD facilitation in Aβ_1–42_-injected rats. **A** Experimental scheme: Rats received i.c.v. injection of soluble Aβ_1–42_ (10 μL each side, 75 μM) under ketamine/xylazine anesthesia. Two weeks after single i.c.v. injection of soluble Aβ_1-42_, the animals were re-anaesthetized with urethane and in vivo electrophysiology (EP) experiments were performed. **B** Application of a peri-threshold weak LFS (bar, LFS-300; 300 high-intensity pulses at 1 Hz) induced robust and stable LTD in Aβ_1–42_-injected rats. One hour post systemic administration of the selective mGluR5 antagonist MTEP (hash; 3 mg/kg, i.p.) completely prevented the induction of LTD by LFS-300 in animals injected i.c.v. with soluble Aβ_1–42_. In contrast, systemic injection of the NMDAR competitive antagonist CPP (hash;10 mg/kg, i.p.) did not affect the induction of LTD by LFS-300 in animals injected i.c.v. with soluble Aβ_1–42_. As summarized in (**C**), the EPSP at 1.5 h measured 71.9 ± 3.7% in Aβ_1–42_ + Veh group (*n* = 4, *P* = 0.0019 compared with Pre, paired *t*), 95.5 ± 5.8% in Aβ_1–42_ + MTEP group (*n* = 5, *P* = 0.6185 compared with Pre and *P* = 0.0171 compared with Aβ_1-42_ + Veh group; paired *t* and one-way ANOVA) and 68.6 ± 5.8% in Aβ_1–42_ + CPP group (*n* = 4, *P* = 0.0092 compared with Pre, and *P* > 0.9999 compared with Aβ_1–42_ + Veh group; paired *t* and one-way ANOVA). Calibration bars for EPSP traces: vertical, 2 mV; horizontal, 10 ms.

Two weeks after single i.c.v. injection of water-soluble synthetic Aβ_1–42_ in vivo electrophysiology experiments were performed in re-anesthetized rats (Fig. 1A). Synaptic transmission was measured in the stratum radiatum of CA1 area of the dorsal hippocampus following stimulation delivered to the Schaffer collateral-commissural pathway. The application of a peri-threshold relatively weak LFS of 300 high-intensity pulses at 1 Hz (LFS-300) did not induce persistent depression in naïve control rats (Fig. S1). In contrast, the same conditioning stimulation induced robust and stable LTD in rats that received i.c.v. injection of water-soluble synthetic Aβ_1–42_ two weeks prior to testing (Fig. 1B, C). Intriguingly systemic administration of the selective mGluR5 antagonist MTEP blocked the Aβ-facilitated LTD. Contrary to MTEP, the NMDAR antagonist CPP, at a dose (10 mg/kg, i.p.) that completely blocks HFS-induced LTP [37], did not affect the induction of the Aβ-facilitated LTD (Fig. 1B, C).

### ISRIB restores LTD facilitation at CA3-to-CA1 synapses in vivo in exogenous Aβ-injected rats

**Fig. 2:**
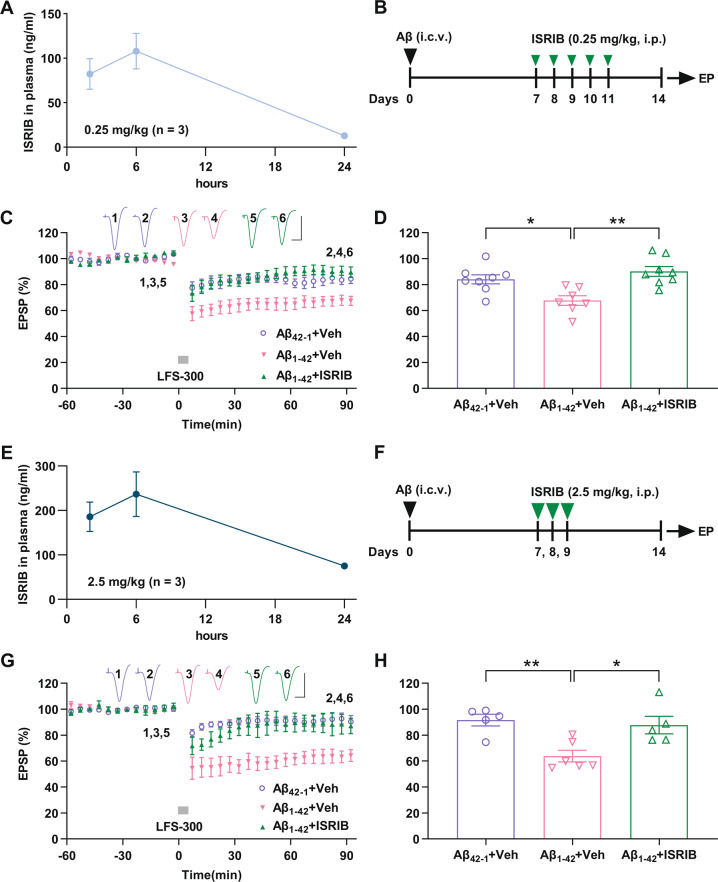
The integrated stress response inhibitor ISRIB reverses facilitation of LTD in Aβ_1–42_-injected rats. **A** Plasma concentration (ng/ml) of ISRIB were determined by high-performance liquid chromatography (HPLC) 2, 6, and 24 h after a single intraperitoneal injection (0.25 mg/kg) in rats (*n* = 3). **B** Experimental scheme: Starting one week after single i.c.v. injection of synthetic Aβ_1–42_ or reverse sequence control peptide Aβ_42–1_ (10 μL each side) rats received a systemic injection of ISRIB (0.25 mg/kg, i.p.) or vehicle for 5 consecutive days. Three days after the final injection of ISRIB, in vivo electrophysiology (EP) experiments were performed under anesthesia. **C** Whereas the application of LFS-300 induced small LTD in control peptide Aβ_42–1_-injected rats, the same conditioning stimulation trigged a robust and persistent LTD in Aβ_1–42_-injected rats. Treatment of ISRIB successfully reversed LTD facilitation in Aβ_1–42_-injected rats. As summarized in (**D**) at 90 min, the EPSP measured 84.1 ± 3.5% in Aβ_42–1_ + Veh (*n* = 8), 67.8 ± 3.7% in Aβ_1–42_ + Veh group (*n* = 7, *P* = 0.0167 compared with Aβ_42–1_ + Veh group; one-way ANOVA) and 90.1 ± 3.8% in Aβ_1–42_ + ISRIB (*n* = 8, *P* = 0.0011 compared with Aβ_1–42_ + Veh and *P* = 0.7370 compared with Aβ_42–1_ + Veh group; one-way ANOVA). **E** Plasma concentration (ng/ml) of ISRIB were determined by HPLC 2, 6, and 24 h after a single intraperitoneal injection (2.5 mg/kg) in rats (*n* = 3). **F** Experimental scheme: Rats were allowed to recover for one week after single i.c.v. injection of synthetic Aβ_1–42_ or reverse sequence control peptide Aβ_42–1_ (10 μL each side) and received an injection of a higher dose of ISRIB (2.5 mg/kg, i.p.) or vehicle for 3 consecutive days. Five days after the final injection of ISRIB, in vivo electrophysiology experiments were performed under anesthesia of urethane. **G** Application of LFS-300 induced robust and persistent LTD in Aβ_1–42_-injected rats. Treatment of ISRIB successfully reversed LTD facilitation in Aβ_1–42_-injected rats. As summarized in (**H**) at 90 min, the EPSP measured 91.6 ± 4.5% in Aβ_42–1_ + Veh (*n* = 5), 63.8 ± 4.5% in Aβ_1–42_ + Veh group (*n* = 6, *P* = 0.0069 compared with Aβ_42–1_ + Veh group; one-way ANOVA) and 87.7 ± 6.8% in Aβ_1–42_ + ISRIB (*n* = 5, *P* = 0.0188 compared with Aβ_1–42_ + Veh and *P* > 0.9999 compared with Aβ_42–1_ + Veh group; one-way ANOVA). Calibration bars for EPSP traces: vertical, 2 mV; horizontal, 10 ms.

Having found the facilitation of LTD in a delayed rat model by single i.c.v. injection of synthetic Aβ_1–42_, we investigated the potential protective effect of ISRIB on the disruption of LTD. Most previous studies on ISRIB have been carried out in mice, where it has been reported to have favorable pharmacokinetic properties after systemic administration. The doses of ISRIB at 0.25 mg/kg and 2.5 mg/kg were commonly used in mice [14, 17, 18, 24, 32–34]. To determine whether ISRIB has similar pharmacokinetic properties in rats, two groups of rats received a single intraperitoneal injection of 0.25 mg/kg and 2.5 mg/kg respectively. The concentration of ISRIB in plasma was measured at 2, 6 and 24 h after injection using HPLC. We found that ISRIB displayed similar plasma kinetics in rats to what has been reported in mice [18, 34] (Fig. 2A, E). Thus, these two doses of ISRIB were chosen for our experiments in this study in rats.

One week after single i.c.v. injection of synthetic Aβ_1–42_ or reverse sequence control peptide Aβ_42–1_, rats received systemic injection of ISRIB (0.25 mg/kg, i.p.) or vehicle for five days, as reported in mice [34] and a similar ISRIB treatment paradigm in our MWM study (see below). The animals were re-anaesthetized with urethane three days after the final injection of ISRIB and in vivo electrophysiology experiments were performed (Fig. 2B). The application of LFS-300 did not induce persistent depression in Aβ_42-1_-injected rats (i.e., rats that had been injected i.c.v. with the reverse sequence peptide Aβ_42–1_) but induced robust and stable LTD in rats that received i.c.v. injection of water-soluble synthetic Aβ_1–42_ (Fig. 2C, D). Significantly, systemic administration of ISRIB blocked Aβ_1–42_-facilitated LTD (Fig. 2C, D).

We also treated another cohort with the higher dose of ISRIB (2.5 mg/kg) [17, 23, 24, 32, 33] for three days, and in vivo electrophysiology experiments were performed five days after the final injection (Fig. 2F). Similar to the lower dose, fewer injections of the higher dose of ISRIB prevented LTD facilitation by Aβ effectively (Fig. 2G, H).

### ISRIB abrogates spatial memory deficits in exogenous Aβ-injected rats

Recent growing evidence suggests that synaptic LTD is a bona fide hippocampal learning and memory mechanism [9, 38, 39] and soluble Aβ-facilitated LTD may underlie learning and memory deficits in early AD [6, 7]. Having found that systemic administration of ISRIB successfully prevented LTD facilitation in rats injected with soluble synthetic Aβ_1–42_, we next determined if it impacted Aβ-induced hippocampus-dependent memory and learning impairments.

**Fig. 3:**
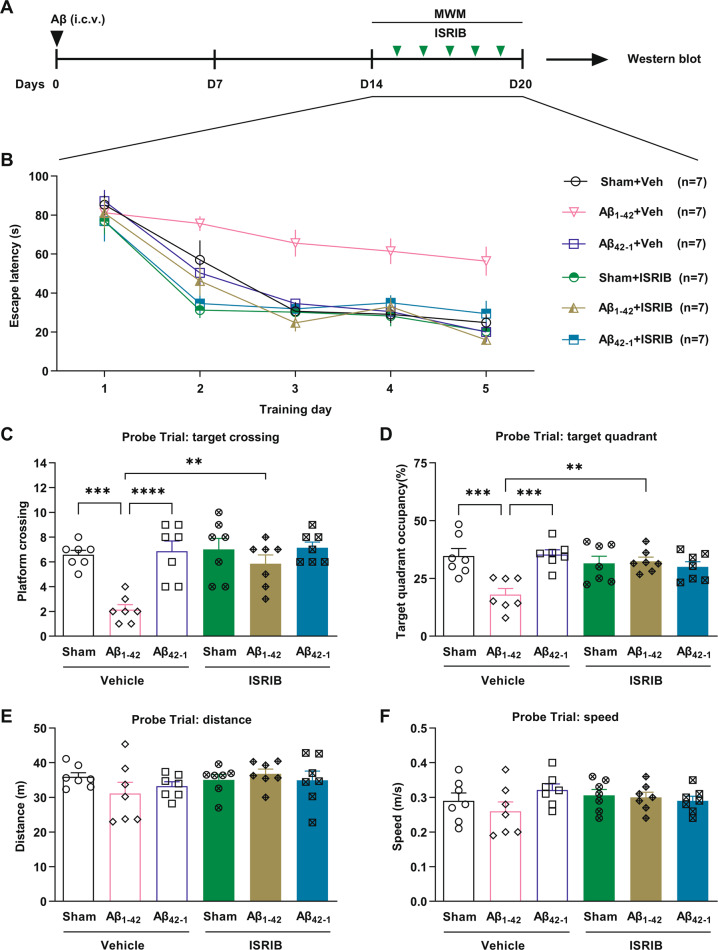
Promotion of learning and memory by ISRIB in Aβ_1–42_-injected rats using a standard MWM protocol. **A** The timeline of experimental design. Water maze training (4 trials per day for 5 days) was performed 2 weeks after i.c.v. injection of Aβ_1-42_ or reverse control Aβ_42–1_. Vehicle (1% DMSO in saline) or ISRIB (0.25 mg/kg, i.p.) were injected immediately after the last training trial in the MWM every day. **B** Escape latency in the navigation trial plotted against the training days. Two-way ANOVA followed by a post hoc Bonferroni multiple comparison test, *P* < 0.0001, *F*_5,36_ = 15.38 (*n* = 7 rats per group). During training, compared with the Aβ_42–1_ + Veh and Sham+Veh group, the Aβ_1–42_ + Veh group spent more time to escape to the hidden platform from day 3 (Aβ_1–42_ + Veh versus Sham + Veh: *P* = 0.0079 on day 3, *P* = 0.0101 on day 4, *P* = 0.0229 on day 5; Aβ_1–42_ + Veh versus Aβ_42–1_ + Veh: *P* = 0.0165 on day 3, *P* = 0.0118 on day 4, *P* = 0.0107 on day 5) but not in the first 2 days. However, a large reduction in escape latency was caused by ISRIB in rats injected with Aβ_1–42_ from day 2 (Aβ_1-42_ + Veh versus Aβ_1-42_ + ISRIB: *P* = 0.0362). **C** In the probe trial (*n* = 7 rats per group), Aβ_1–42_ + Veh animals appeared to cross the platform less frequently compared with the Aβ_42–1_ + Veh and Sham + Veh group and ISRIB significantly improved performance (*P* = 0.0001, one-way ANOVA followed by a post hoc Bonferroni multiple comparison test). **D** In the case of the probe trial quadrant bias, ISRIB significantly enhanced target quadrant occupancy in the Aβ_1–42_-injected animals (Aβ_1–42_ + Veh versus Aβ_1–42_ + ISRIB: *P* = 0.0020, one-way ANOVA followed by a post hoc Bonferroni multiple comparison test). **E**, **F** Both total swimming distance (*P* = 0.4351, one-way ANOVA) and swimming speed (*P* = 0.3626, one-way ANOVA) are comparable in all the groups. Error bars, s.e.m.

Water maze training with a standard protocol (four trials per day) was started 2 weeks after i.c.v. injection of Aβ_1-42_ or reverse control Aβ_42–1_. ISRIB (0.25 mg/kg, i.p.) or vehicle was injected immediately after the last training trial every day [18] (Fig. 3A). Whereas repeated training caused a day-to-day decrease in escape latency in the sham surgery group or Aβ_42–1_ injected group, Aβ_1–42_ inhibited the acquisition of the spatial task, with a more gradual learning curve slope/longer escape latencies. ISRIB treatment consistently significantly shortened escape latencies in Aβ_1–42_-injected rats from day 2 (Fig. 3B). Aβ_1–42_-injected rats crossed the platform area much less compared with sham surgery rats and the Aβ_42–1_ injected group when the platform was removed 24 h after the last training trial and ISRIB significantly reversed the memory deficit caused by Aβ_1–42_ injection (Fig. 3C). We also observed that Aβ_1–42_-injected rats spent much less time in the target quadrant compared with the control groups in the probe trial and ISRIB treatment restored recall to normal (Fig. 3D). Both the total swim distance (Fig. 3E) and swim speed (Fig. 3F) were comparable in all the groups, which indicates that general movement ability was not affected.

**Fig. 4:**
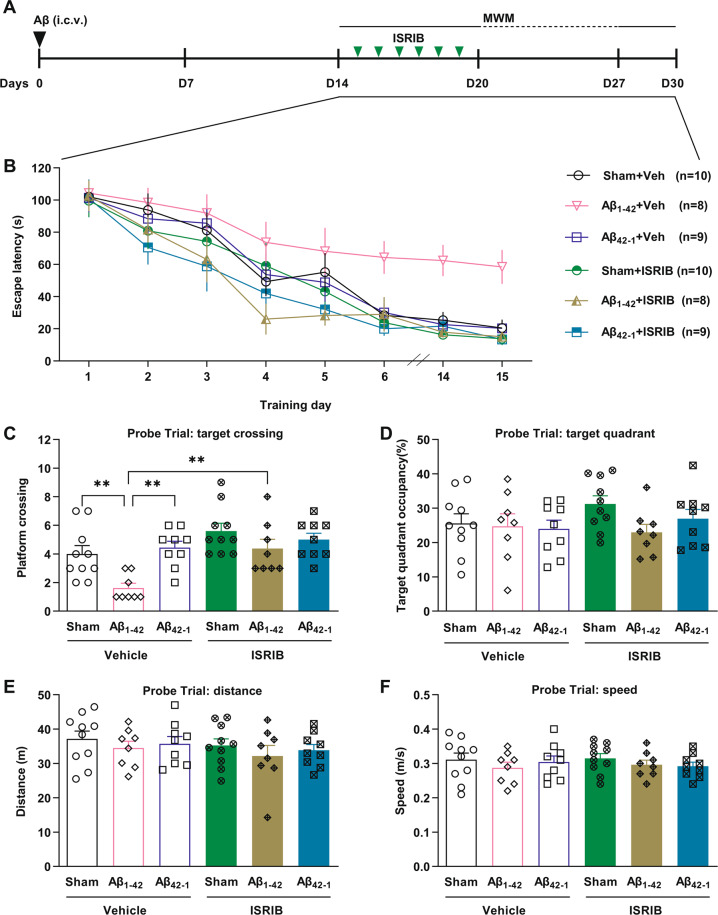
Spatial learning and memory deficits in Aβ_1–42_-injected rats are abrogated by ISRIB using a weak MWM training protocol. **A** The timeline of experimental design. All animals were trained with 1-trial/day. Vehicle or ISRIB (0.25 mg/kg, i.p.) were injected immediately after the training session in the MWM every day. **B** All the rats spent less time gradually to find the hidden platform after each training trial. Aβ_1–42_-injected rats spent more time to find the hidden platform from day 6 and on day 14 and day 15 after one-week break (*n* = 8–10 rats per group, Two-way ANOVA followed by a post hoc Bonferroni multiple comparison test, *P* < 0.0001, *F*_5,48_ = 8.778. Aβ_1–42_ + Veh versus Sham+Veh: *P* = 0.0681 on day 6, *P* = 0.0325 on day 14, *P* = 0.0415 on day 15; Aβ_1–42_ + Veh versus Aβ_42–1_ + Veh: *P* = 0.1142 on day 6, *P* = 0.0207 on day 14, *P* = 0.0410 on day 15) and ISRIB significantly improved performance (Aβ_1–42_ + Veh versus Aβ_1–42_ + ISRIB: *P* = 0.0146 on day 6, *P* = 0.0231 on day 14, *P* = 0.0158 on day 15). No difference was detected among Sham + Veh, Aβ_42–1_ + Veh, Sham + ISRIB, Aβ_42–1_ + ISRIB and Aβ_1–42_ + ISRIB groups (Repeated measures ANOVA. *P* = 0.0569, *F*_4,41_ = 2.504). **C** In the probe trial (*n* = 8–10 rats per group), Aβ_1–42_-injected animals crossed the platform much less compared with control groups (Aβ_1–42_ + Veh versus Sham + Veh: *P* = 0.0129; Aβ_1–42_ + Veh versus Aβ_42–1_ + Veh: *P* = 0.0028) and ISRIB significantly enhanced platform crossing in Aβ_1–42_-injected rats (Aβ_1–42_ + Veh versus Aβ_1–42_ + ISRIB: *P* = 0.0050, One-way ANOVA followed by a post hoc Bonferroni multiple comparison test). **D**–**F** All the groups were similar in target quadrant occupancy (*P* = 0.9409, One-way ANOVA) (**D**) and total swimming distance (*P* = 0.7056, One-way ANOVA) (**E**) and swimming speed (*P* = 0.7876, One-way ANOVA) (**F**). Error bars, s.e.m.

Previously, ISRIB showed promising memory-enhancing effects in wild-type animals trained in the MWM with a weak protocol consisting of one swimming trial per day [18]. Here, having not seen consistent memory enhancement by ISRIB in control animals trained in the MWM with a standard protocol, we performed navigation training with a weak protocol (1 trial per day) in rats 2 weeks after i.c.v. injection of Aβ_1–42_ or reverse control Aβ_42–1_ or age-matched rats with sham surgery. The same dose of ISRIB was injected immediately after the training session every day (Fig. 4A). Having found that daily injection of ISRIB for six days improved escape latency in Aβ_1–42_-injected rats (Fig. 4B), we wondered if the beneficial effects persist after ceasing treatment [40]. Indeed, one week after stopping its administration ISRIB still significantly shortened escape latency in Aβ_1–42_-injected rats (Fig. 4B). Although ISRIB significantly restored learning ability on days 6, 14, and 15 in animals that had been injected i.c.v. with Aβ_1–42_, this small molecule only caused a relatively weak and transient enhancement in sham surgery rats and Aβ_42–1_-injected rats on days 2–5 (Fig. 4B). Aβ_1–42_ also caused a memory deficit, which was reversed by ISRIB, in the probe trial as measured by platform location crossings (Fig. 4C). However, the target quadrant occupancy was comparably poor in all the groups during the probe trial (Fig. 4D). The apparent difference in target quadrant occupancy between the control groups receiving the one trial a day training protocol (Fig. 4D) compared with the standard four trials per day protocol (Fig. 3D) is likely a consequence of the reduced total number of training trials and/or the one-week training gap in the former group. Neither i.c.v. injection of Aβ peptide nor systemic injection of ISRIB affected the movement ability of rats as measured by swimming distance or speed (Fig. 4E, F). These results, together with four trials a day study data, support the ability of ISRIB to abrogate learning and memory deficits caused by Aβ_1–42_.

### ISRIB restores aberrant protein synthesis in exogenous Aβ-injected rats

**Fig. 5:**
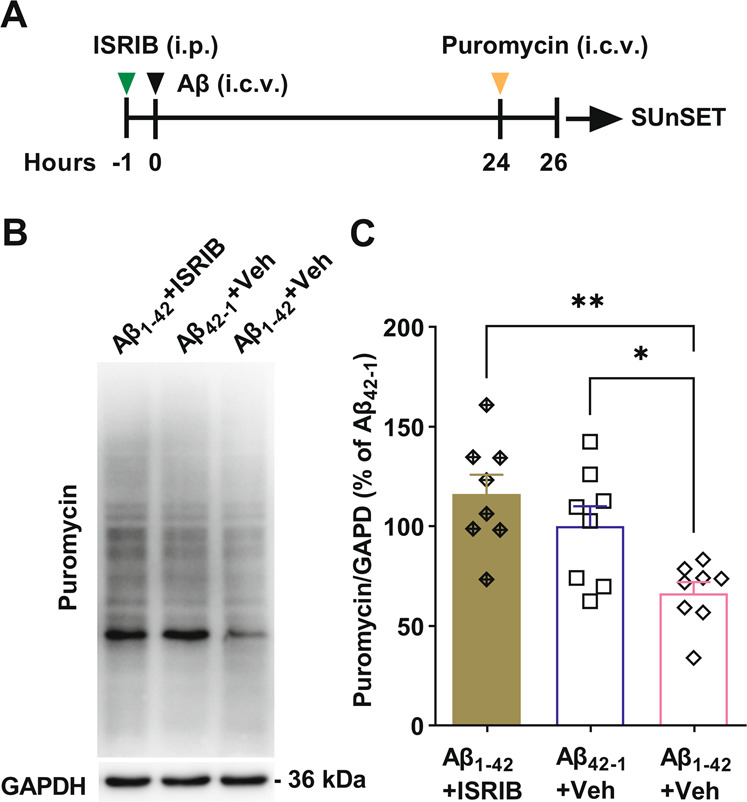
ISRIB prevented the disruption of protein synthesis induced by Aβ_1–42_. **A** Experimental timeline: Rats received systemic and i.c.v. injections under isoflurane anesthesia. ISRIB (2.5 mg/kg, i.p.) was administrated 1 h before Aβ injection (i.c.v., 10 μL each side). Rats were re-anesthetized 24 h after Aβ injection with isoflurane and received an i.c.v. injection of puromycin (5 μL each side, 10 μg/μL). Brain samples were collected 2 h after puromycin injection. **B** Protein extracts were separated by electrophoresis and analyzed by western blot with antibody to puromycin. GAPHD immunoblot is shown as a loading control (bottom). **C** Levels of newly synthesized proteins labeled with puromycin were significantly lower in Aβ_1–42_ + Veh group compared with Aβ_42–1_ + Veh group (*n* = 8, *P* = 0.0386, one-way ANOVA). In ISRIB treatment group, protein synthesis was markedly restored (*n* = 8, *P* = 0.0018, Aβ_1–42_ + ISRIB compared with Aβ_1–42_ + Veh; *P* = 0.6087, Aβ_1–42_ + ISRIB compared with Aβ_42–1_ + Veh group; one-way ANOVA). Error bars, s.e.m.

Protein synthesis provides a mechanism for the persistence of memory and is important in the late phase of many forms of synaptic plasticity including LTD [41]. Elevated eIF2α phosphorylation has been observed in most Aβ-related animal AD models (but see [42, 43]) and eIF2α phosphorylation suppresses general protein synthesis. We investigated the expression level of phosphorylated eIF2α in western blots of hippocampal tissue from the rats that received an exogenous Aβ injection in the four training trials a day MWM study (Fig. S2A). As expected, the level of eIF2α phosphorylation was significantly increased in the hippocampus of Aβ_1–42_-injected rats (Figs. S2B and S3). The control reverse peptide, Aβ_42–1_, also slightly, although not significantly, increased eIF2α phosphorylation. This indicates that a single i.c.v. injection of an exogenous peptide may have subtle persistent unspecific effects on hippocampal biochemistry. Surprisingly, ISRIB treatment reduced p-eIF2α level in Aβ_1-42_-injected animals (Figs. S2B and S3), suggesting that ISRIB may reset ISR activation as reported recently [24]. To determine whether single i.c.v. injection of Aβ suppresses general protein synthesis and whether ISRIB can correct translation defects in live rats, we modified the in vivo SUnSET [35, 36] to assess de novo protein synthesis 24 h after single i.c.v. injection of Aβ with or without pre-treatment of ISRIB (Fig. 5A). Aβ_1–42_ injection caused a significant repression of general protein synthesis and treatment with ISRIB completely prevented this decrease (Fig. 5B, C and Fig. S4). Phosphorylation of eIF2α preferentially enhances translation of some mRNAs such as ATF4 which plays an important role in synaptic plasticity and memory [16]. We, therefore, investigated the expression level of ATF4 in the hippocampal tissue from the rats that received an exogenous Aβ injection in the 4 training trials a day MWM study (Fig. S2A). Aβ_1–42_ injection caused a significant increase in ATF4, which ISRIB completely reversed (Figs. S2C,S3).

## Discussion

We report here that soluble Aβ causes ISR induction in the promotion of LTD mechanisms in the dorsal hippocampus in vivo. Remarkably, treatment with ISRIB, completely prevented LTD facilitation. Therefore, it seems likely that the restoration of normal protein synthesis by ISRIB prevents Aβ-facilitated LTD. This finding is consistent with prior evidence that certain forms of LTD, in particular mGluR-dependent LTD, require eIF2α phosphorylation [14–17]. mGluR-LTD was reported to be prevented by either genetically reducing eIF2α phosphorylation or pharmacologically suppressing phospho-eIF2α controlled translation with ISRIB. By contrast, increased eIF2α phosphorylation by eIF2α phosphatase inhibitor Sal300 facilitates mGluR-LTD [14]. Interestingly, mGluR-LTD is enhanced under pathophysiological conditions such as Fragile X syndrome models [10] and AD Aβ [11].

Although the cellular mechanisms of hippocampal mGluR-LTD have been well documented, little is known about the contribution of this form of plasticity to hippocampal-dependent learning. Abdou et al. reported that induction of NMDAR-dependent LTD at synapses in engram cell assemblies can erase memory, indicating the close relationship between LTD and forgetting [44]. Since the balance between forgetting and memory consolidation is crucial under physiological conditions [45], normal LTD is a bona fide learning and memory mechanism. Optogenetic activation of hippocampal memory engram cells results in memory retrieval in amnesic mouse models of early AD [46], which indicates that elevated forgetting rather than impaired memory formation causes memory deficits in early AD. Aβ-facilitated mGluR-LTD may underly the mechanisms of enhanced forgetting which contributes to the amnesia in early AD. Despite the shortage of direct evidence between excessive mGluR-LTD and elevated forgetting in AD, the finding that hippocampal mGluR-LTD are altered in animal models of AD has led to novel therapeutics for this disease acting at mGluR5. Indeed, chronic administration of the orally bioavailable mGluR5-selective negative allosteric modulator CTEP reverses cognitive decline in the APPswe/PS1 transgenic mice and reduces Aβ plaque deposition [13]. Another study found that the mGluR5 silent allosteric modulator BMS-984923 effectively rescues memory deficits in APPswe/PS1 mice and prevents synaptic dysfunction in Aβ oligomer-treated brain slices and APPswe/PS1 mice [47]. Although ISRIB provides promising protective effects in our Aβ-facilitated LTD model and Aβ-induced spatial learning and memory deficit, whether this enhancement in LTD leads to the spatial memory deficit directly still needs to be elucidated.

De novo protein synthesis-dependent synaptic plasticity is a likely critical step required for the generation of long-term memories. Consistent with an important role for the ISR in mediating Aβ-mediated persistent disruption of synaptic learning mechanisms, we found that ISRIB restored aberrant decrease of protein synthesis and abrogated a learning and memory deficit caused by synthetic Aβ_1–42_ in the water maze.

Growing evidence indicates that eIF2α phosphorylation which is tightly regulated by four kinases (HRI, PKR, PERK, and GCN2) is a memory suppressor. Either reduction of eIF2α phosphorylation or deletion/inhibition of the expression of any of the eIF2α kinases in the brain enhances memory in a variety of behavioral tasks [48–52]. Conversely, increasing eIF2α phosphorylation, even when restricted to CA1 pyramidal neurons, impairs hippocampal memory consolidation [53], suggesting that specific translational changes downstream of eIF2α phosphorylation are required for memory regulation.

Aberrant elevated phospho-eIF2α has been found in sporadic AD patients’ brains [34, 54–59] and in different transgenic mouse models of AD, including APP/PS1 [34, 60, 61], Tg2576 [56, 62] and 5XFAD [57, 62, 63], (but see [43]). In the present study, post-mortem examination of the brains indicated that eIF2α phosphorylation and ATF4 were elevated by Aβ_1–42_ and ISRIB appeared to reduce ATF4 and eIF2α phosphorylation levels. These findings are consistent with evidence that the addition of oligomeric Aβ_1–42_ induced aberrant expression of mRNAs of ATF4 [34, 64, 65] and the known mechanism of action of ISRIB. ISRIB reverses the attenuation of the guanine nucleotide exchange factor eIF2B by p-eIF2α [20, 21]. Although ISRIB works downstream of eIF2α phosphorylation, Krukowski et al. recently discovered that ISRIB treatment reduced p-eIF2α levels in aged mice brains via breaking a feedback loop of ATF4-GADD34-eIF2α phosphatase, thereby resetting age-related ISR activation [24]. Whether or not ISRIB can reset Aβ-induced ISR activation in our Aβ_1–42_-injected rat model needs to be elucidated. ISRIB, unlike the PERK inhibitor GSK2606414, only partially restores protein synthesis and confers neuroprotection without adverse effects on the pancreas most probably due to its state-dependent action [23, 25, 40, 66]. Restoration of protein synthesis by ISRIB has been reported in different nervous system disease models [33, 67, 68] including prion-diseased mice [23], primary cortical neurons from Aβ-depositing APPSwe transgenic mice [69], and Aβ-treated mouse hippocampal slices [34].

ATF4 is a key regulator for hippocampal long-term synaptic plasticity and memory formation [16] and its expression level can be paradoxically upregulated by phosphorylation of eIF2α which leads to the inhibition of general protein synthesis. The protein level of ATF4 is increased in the cortex of AD brains [64, 70] and the increased translation level of ATF4 in axons may mediate the spread of AD pathology [64]. ATF4 also binds to the regulatory region of the human presenilin-1 gene and therefore is critical for gamma-secretase activity which in turn promotes the production of Aβ [71]. Our finding that ISRIB appeared to restore elevated levels of ATF4 protein caused by Aβ is consistent with reports that ISRIB blocks the production of ATF4 upon GCN2 or HRI activation [18, 34, 72]. However, whether restoration of ATF4 mediated the protective effects of ISRIB in our Aβ-injected rat model is not clear and further investigation is needed.

Apart from endoplasmic reticulum stress caused by the unfolded protein response, other Aβ-mediated AD pathologies including glutamate excitotoxicity, hypoxia, and neuronal inflammation can also induce the ISR [1, 2]. Both Aβ-containing AD brain extracts and purified Aβ dimers potentially suppress glutamate reuptake and subsequently induce neuronal hyperactivation [73]. Hypoxia with decreased cerebral blood flow has been found early in AD and a body of evidence indicates that Aβ has vasoactive and vasculotoxic effects on blood vessels, in particular, capillaries at pericyte locations [74].

Some reports indicate that ISRIB is not effective in certain transgenic APP and tau mouse models, possibly because of ISRIB’s pharmacological profile or differences in the level of engagement of the ISR in these models [42, 43, 69, 75, 76]. The very high failure rate of AD clinical trials may be partly due to the premature translation of successful pathology reduction in transgenic mice to humans [77]. Thus, choosing appropriate models in AD research is extremely important [78]. Our findings are in line with very recent findings from mice that ISRIB restores hippocampal protein synthesis and corrects synaptic plasticity disruption and learning and memory deficits [34]. Compared with transgenic models, whether or not animal models incorporating injected soluble Aβ here more closely mimic key early pathological changes in sporadic AD patients need to be carefully addressed in future studies.

## Conclusions

In summary, the small-molecule ISRIB provides promising protective effects on our Aβ-facilitated LTD model and Aβ-induced spatial learning and memory deficit. The beneficial action of ISRIB may be mediated by its ability to restore Aβ-induced aberrant protein synthesis including ATF4 elevation in the hippocampus. Targeting the ISR by suppressing the eIF2α phosphorylation cascade with ISRIB may provide protective effects against the synaptic and cognitive disruptive effects of Aβ in the early stage of sporadic AD.

## Supplementary information

Supplementary information

## Data Availability

All data supporting this study are available from the corresponding author upon reasonable request.
