# Supplementary material for: Inhibition of the ISR abrogates mGluR5-dependent long-term depression and spatial memory deficits in a rat model of Alzheimer’s disease
Source: Transl Psychiatry. 2022 Mar 8;12:96. doi: 10.1038/s41398-022-01862-9 (PMC8904583; doi:10.1038/s41398-022-01862-9)
Supplement: Supplementary file 1 — Supplementary information [file 41398_2022_1862_MOESM1_ESM.pdf]

Inhibition of the ISR abrogates mGluR5-dependent long-term depression and spatial memory deficits in a rat model of Alzheimer's disease

Zhengtao Hu, Pengpeng Yu, Yangyang Zhang, Yin Yang, Manyi Zhu, Shuanying Qin, Ji-Tian Xu, Dongxiao Duan, Yong Wu, Deguo Wang, Michael J. Rowan and Neng-Wei Hu

Supplementary Figures:

Supplementary Figure S1. Peri-threshold low-frequency stimulation failed to induce long-term depression at CA3-to-CA1 synapses *in vivo*.

Supplementary Figure S2. Effects of $A\beta_{1-42}$ and ISRIB on p-eIF2α and ATF4 levels.

Supplementary Figure S3. Full Western blots of p-eIF2α and ATF4 obtained in this study.

Supplementary Figure S4. Full Western blots of SUnSET obtained in this study.

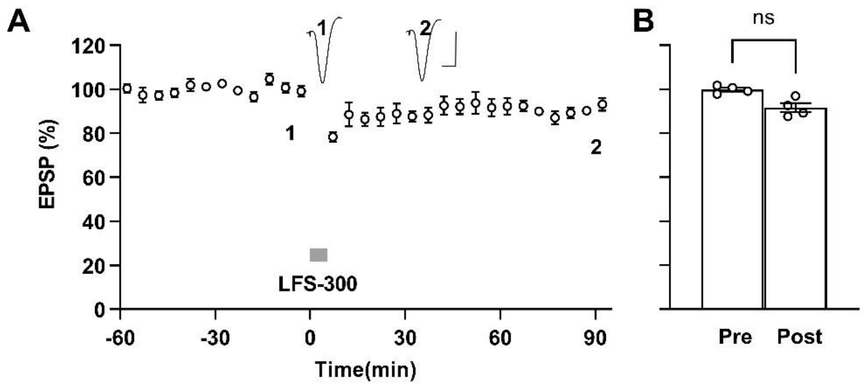

Figure S1. Peri-threshold low-frequency stimulation failed to induce long-term depression at CA3-to-CA1 synapses *in vivo*. (**A**) Application of a peri-threshold weak LFS (bar, LFS-300; 300 high-intensity pulses at 1 Hz) did not induce obvious LTD in naïve control rats. As summarized in (**B**), the EPSP at 90 min measured 91.7 ± 2.1% (n = 4, P = 0.0569 compared with Pre, paired t). Calibration bars for EPSP traces: vertical, 2 mV; horizontal, 10 ms.

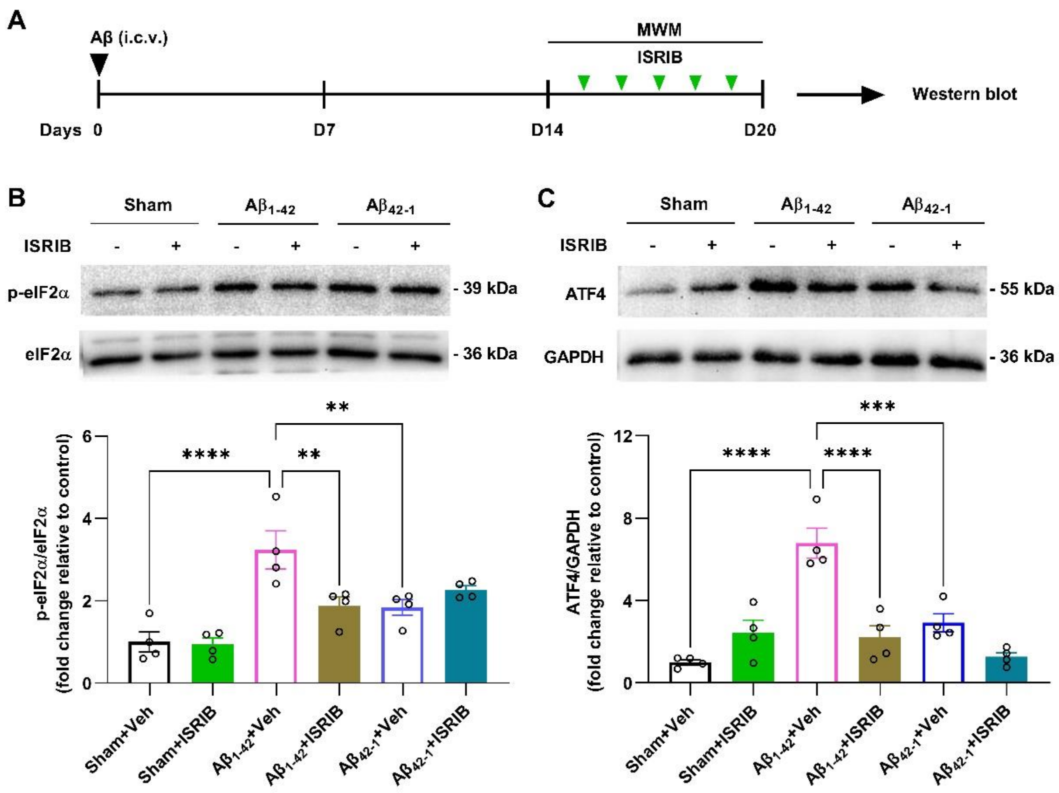

Figure S2. Effects of $A\beta_{1-42}$ and ISRIB on p-eIF2α and ATF4 levels.

(**A**) The timeline of experimental design. The expression levels of p-eIF2α and ATF4 were assayed in the hippocampal tissue from the rats in figure 3. (**B**) Western blots showing that the level of p-eIF2α was increased in the hippocampus after i.c.v. injection of $A\beta_{1-42}$ (n = 4, $P < 0.0001$, $A\beta_{1-42}$+Veh compared with Sham+Veh group; one-way ANOVA) while the injection of the reverse sequence peptide $A\beta_{42-1}$ did not obviously change the level of p-eIF2α (n = 4, $P = 0.1966$, $A\beta_{42-1}$+Veh compared with Sham+Veh group; one-way ANOVA). Treatment of ISRIB (0.25 mg/kg, i.p.) reduced the levels of p-eIF2α in $A\beta_{1-42}$-injected rats (n = 4, $P = 0.0083$, $A\beta_{1-42}$+Veh compared with $A\beta_{1-42}$+ISRIB group; one-way ANOVA). (**C**) The level of ATF4 increased in $A\beta_{1-42}$-injected rats (n = 4, $P < 0.0001$, compared with Sham+Veh; $P = 0.0002$ compared with $A\beta_{42-1}$+Veh group; one-way ANOVA) but the injection of the reverse sequence peptide $A\beta_{42-1}$ did not change the level of ATF4 (n = 4, $P = 0.0950$, compared with Sham+Veh group; one-way ANOVA). Treatment of ISRIB restored ATF4 to normal level (n = 4, $P < 0.0001$, $A\beta_{1-42}$+Veh compared with $A\beta_{1-42}$+ISRIB; $P = 0.7060$, $A\beta_{1-42}$+ISRIB compared with Sham+Veh group; one-way ANOVA). Error bars, s.e.m.

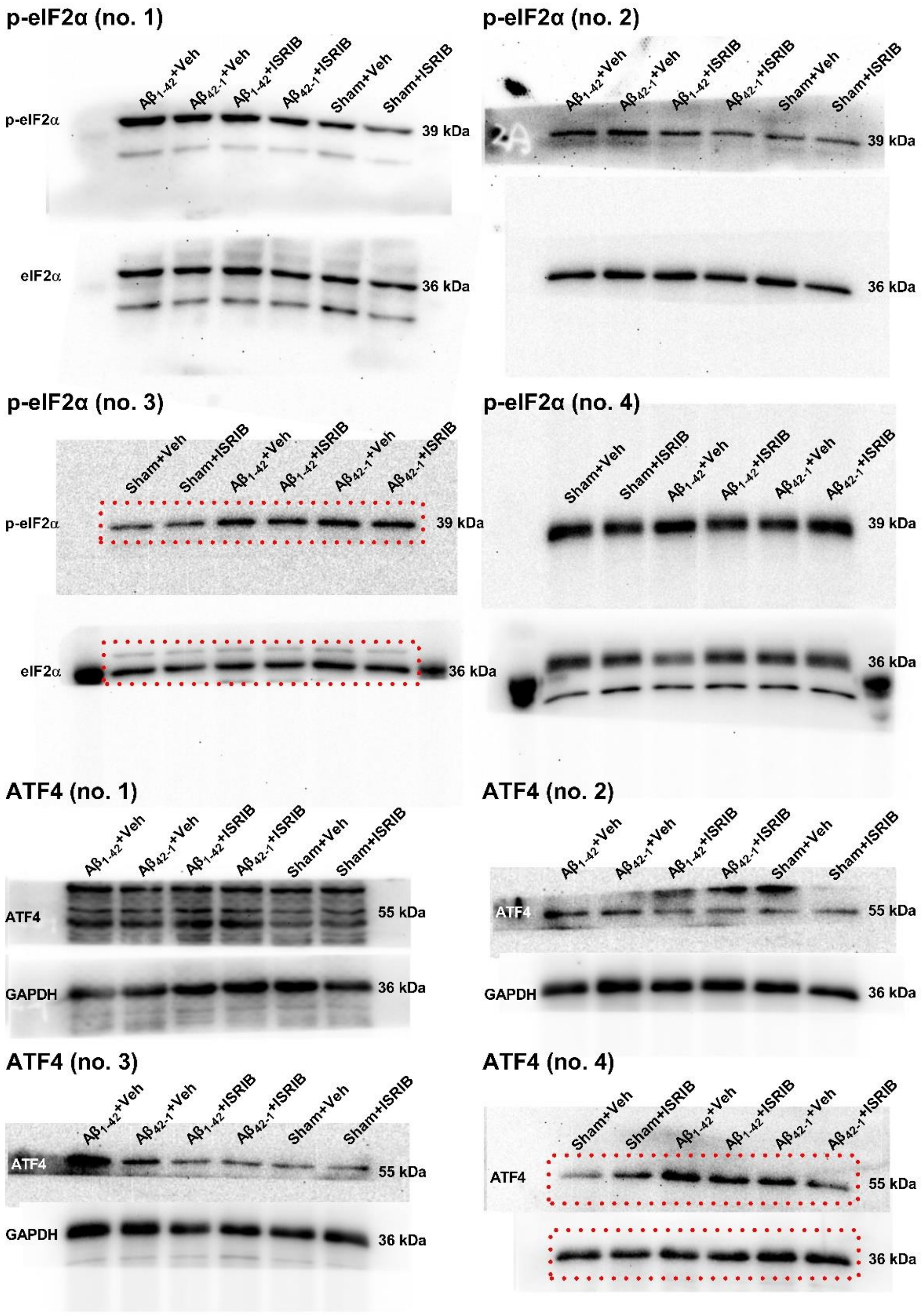

Figure S3. Full Western blots of p-eIF2α and ATF4 obtained in this study. Lanes shown in Figure S2 are boxed in red. Anti-ATF4 antibody for no.1-3: A18687 (1:1000), ABclonal; anti-ATF4 antibody for no.4: ab23760 (1:1000), Abcam.

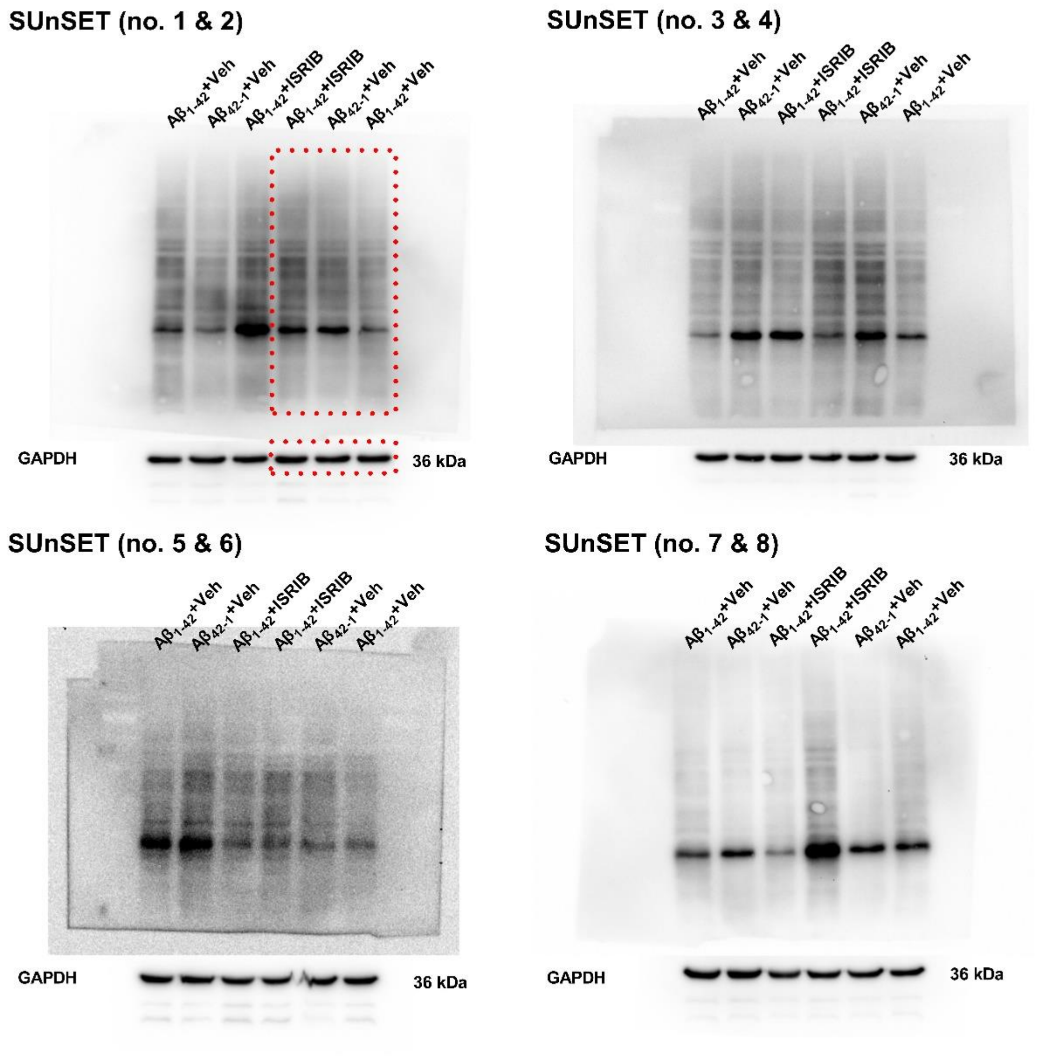

Figure S4. Full Western blots of SUnSET obtained in this study. Lanes shown in Figure 5 are boxed in red.
